# Next-generation Bruton’s tyrosine kinase inhibitors for chronic lymphocytic leukemia-associated membranoproliferative glomerulonephritis: a case report

**DOI:** 10.1007/s00277-026-06730-w

**Published:** 2026-01-30

**Authors:** Li Chen, Yuting Huang, Jing Xu, Sinian Huang, Qianying Zhang, Fanli Hua, Xiaoxia Pan, Jian-Qing Mi

**Affiliations:** 1 Shanghai Institute of Hematology, State Key Laboratory of Medical Genomics, National Research Center for Translational Medicine at Shanghai, Ruijin Hospital Affiliated to Shanghai Jiao Tong University School of Medicine, Shanghai, China; 2 Shanghai Jiao Tong University School of Medicine, Shanghai, China; 3 Department of Nephrology, Ruijin Hospital Affiliated to Shanghai Jiao Tong University School of Medicine, Shanghai, China; 4 Department of Pathology, Qingpu Branch of Zhongshan Hospital, Fudan University, Shanghai, China; 5 Department of Hematology, Qingpu Branch of Zhongshan Hospital, Fudan University, Shanghai, China

**Keywords:** Chronic lymphocytic leukemia, Membranoproliferative glomerulonephritis, Nephrotic syndrome, Bruton’s tyrosine kinase inhibitors, Kidney biopsy

## Abstract

Renal involvement in chronic lymphocytic leukemia (CLL) is uncommon but can lead to significant morbidity. Membranoproliferative glomerulonephritis (MPGN) is among the most frequently reported glomerular lesions associated with CLL and may presents with nephrotic syndrome. Early recognition of the association between renal lesions and CLL is crucial for guiding treatment and improving both renal and hematologic outcomes. We report two biopsy-proven cases of CLL-associated MPGN successfully treated with next-generation Bruton’s tyrosine kinase inhibitors (BTKis). Both patients presented with nephrotic-range proteinuria. In Case 1, the patient exhibited monoclonal IgG-κ gammopathy and isolated low serum C3, suggestive of complement-mediated injury without direct immunoglobulin deposition. He achieved sustained hematologic and renal remission with orelabrutinib following early discontinuation of rituximab–chlorambucil due to infection. In Case 2, renal biopsy showed interstitial infiltration by CLL cells and immune complex deposition, supporting a leukemic infiltration and immune-complex mediated mechanism. Zanubrutinib led to clinical improvement, and rituximab was later added to further reduce proteinuria. These cases underscore the critical role of kidney biopsy in clarifying diagnosis and underlying mechanisms. In this case series, the treatment regimen centered on next-generation BTKis enabled patients to achieve concurrent favorable renal and hematologic remission with good tolerability.

## Introduction

Chronic lymphocytic leukemia/small lymphocytic lymphoma (CLL/SLL) is an indolent B-cell malignancy, often diagnosed incidentally and without an immediate need for treatment in most patients [1]. Although most patients present with bone marrow and lymph node involvement, extramedullary manifestations are not uncommon. However, renal complications remain rare and are frequently under-recognized, with renal biopsies performed in less than 2% of CLL patients [2].

Variety of renal lesions have been reported in association with CLL, reflecting diverse underlying pathogenic mechanisms. These include immune complex–mediated diseases such as membranoproliferative glomerulonephritis (MPGN), membranous nephropathy, minimal change disease (MCD), and crescentic glomerulonephritis, as well as direct leukemic infiltration, thrombotic microangiopathy (TMA), and interstitial nephritis. The pathogenesis is equally heterogeneous, involving monoclonal immunoglobulin deposition, complement pathway dysregulation, cryoglobulinemia, and in some cases, lymphomatous infiltration of renal parenchyma. These conditions may lead to clinical presentations ranging from asymptomatic proteinuria to nephrotic syndrome, acute kidney injury, or even end-stage renal disease, highlighting the indispensable role of renal biopsy in accurate diagnosis and treatment planning [2–6].

However, due to the rarity of such complications and limited biopsy data, evidence regarding optimal management remains sparse. In particular, the potential role of Bruton’s tyrosine kinase inhibitors (BTKis), which are now widely used in CLL, in treating CLL-associated renal manifestations has not been well characterized.

Here, we describe two cases of biopsy-confirmed MPGN in patients with CLL who were treated with next-generation BTKis, either alone or in combination with rituximab under different clinical contexts. Both patients achieved renal and hematologic improvements during therapy. To our knowledge, this is the first report describing rapid and durable renal remission with next-generation BTKis in CLL-associated MPGN.

## Case 1: CLL initially presenting with MPGN in a young patient

In September 2022, a 29-year-old man presented with diarrhea, progressive bilateral lower extremity edema, and oliguria. Laboratory tests (Table 1) revealed lymphocytosis (3.56 × 10⁹/L), moderate anemia (hemoglobin 67 g/L), and thrombocytopenia (43 × 10⁹/L). Peripheral blood flow cytometry identified 62.8% abnormal mature B lymphocytes with the following immunophenotype: CD5⁺, CD10⁻, CD200⁺, CD43⁻, CD23⁻, CD22⁺, CD79b⁻, CD20⁺, FMC7⁻, and negative for both κ and λ light chains—findings consistent with a clonal B-cell population. Additional tests indicated nephrotic syndrome, characterized by massive proteinuria (24-hour urinary protein: 7643 mg; urine albumin-to-creatinine ratio [uACR]: 209.9 mg/mmol), marked hypoalbuminemia (25 g/L), and impaired renal function (serum creatinine: 103 µmol/L; uric acid: 709 µmol/L).

Immunological workup showed isolated low complement C3 (0.68 g/L) with normal C4, and hypogammaglobulinemia (IgG, IgA, IgM all below normal range). Serum immunofixation electrophoresis identified an IgG-κ monoclonal protein (1.2 g/L), and urine tested positive for free κ light chains. Serum β₂-microglobulin was markedly elevated at 6038 ng/mL. Imaging demonstrated generalized lymphadenopathy and splenomegaly (84 × 224 mm). Lymph node biopsy confirmed CLL/SLL. Bone marrow flow cytometry showed abnormal mature B cells comprising 63.4% of lymphocytes, with the following phenotype: CD19⁺, CD5⁺, CD23⁻, CD22⁺, CD200⁺, FMC7⁻, Kappa⁻, Lambda⁻—consistent with CLL/SLL. Fluorescence in situ hybridization (FISH) analysis revealed no abnormalities in TP53 (17p13.1), ATM (11q22.3), trisomy 12, or deletion 13q14 (D13S319/RB1). Sanger sequencing identified unmutated IGHV status. The disease was staged as Rai IV / Binet C, with a high-risk CLL-IPI score of 5.

**Fig. 1:**
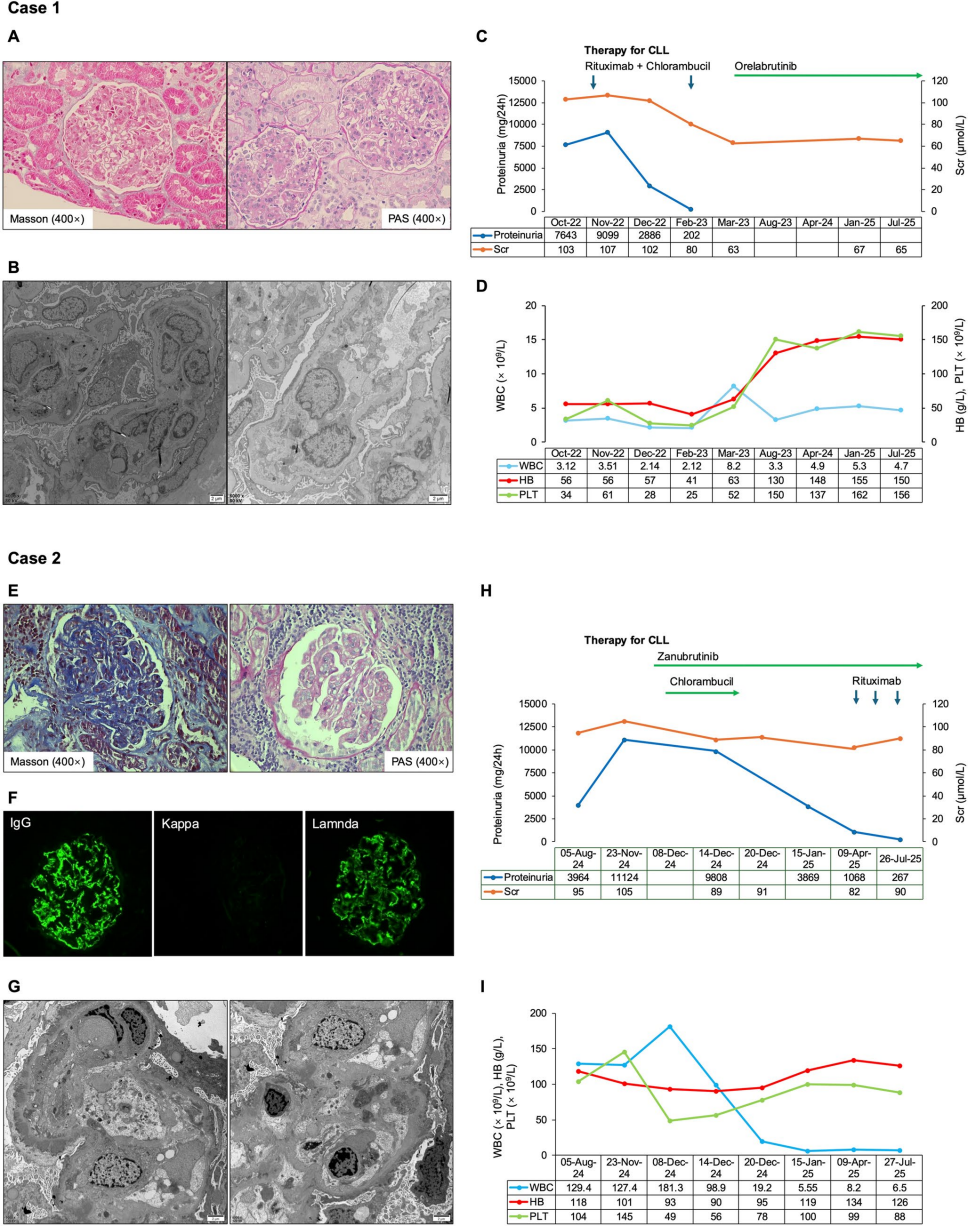
Clinicopathological and therapeutic features of two patients with chronic lymphocytic leukemia (CLL)-associated membranoproliferative glomerulonephritis (MPGN). **A** Light microscopy of kidney biopsy in Case 1 showing mesangial hypercellularity and double-contour formation consistent with MPGN (Masson's trichrome, PAS, × 400) . **B** Electron microscopy of Case 1 kidney biopsy revealing endocapillary proliferation and sparse subendothelial electron-dense deposits. **C** Dynamic changes in serum creatinine and 24-hour proteinuria during treatment in Case 1. **D** Dynamic changes in key hematological parameters in Case 1 during treatment. **E** Light microscopy of kidney biopsy in Case 2 showing mesangial and endocapillary proliferation with double-contour formation (Masson's trichrome, PAS, × 400). **F** Immunofluorescence of Case 2 biopsy demonstrating granular IgG and lambda light chain deposition in glomeruli, with no deposition of kappa light chain. **G** Electron microscopy in Case 2 showing abundant subepithelial and intramembranous electron-dense deposits and sparse subendothelial and mesangial electron-dense deposits. **H** Dynamic changes in serum creatinine and 24-hour proteinuria during treatment in Case 2. **I** Dynamic changes in key hematological parameters in Case 2 during treatment

In November 2022, the patient was enrolled in a clinical trial (CTR20201980) and randomized to the control arm. He received rituximab (375 mg/m² on day 1 of cycle 1; 500 mg/m² on day 1 of cycles 2–6) in combination with chlorambucil (0.5 mg/kg on days 1 and 15 of each 28-day cycle, for 6 cycles), along with valsartan to control proteinuria. After the first treatment cycle, the patient experienced complete resolution of edema, normalization of both serum albumin and creatinine, and a significant reduction in proteinuria (24-hour urinary protein: 202 mg), meeting the criteria for complete remission of nephrotic syndrome, defined as proteinuria < 0.3 g/day according to the 2021 Kidney Disease: Improving Global Outcomes (KDIGO) Clinical Practice Guideline for Glomerular Diseases [7]. However, the patient continued to exhibit persistent cytopenias along with no reduction in lymphadenopathy or splenomegaly, suggesting inadequate hematologic response. A renal biopsy was performed to clarify the underlying cause of the nephrotic syndrome. Histological evaluation revealed diffuse mesangial cell proliferation with capillary loop narrowing and occlusion, segmental endocapillary hypercellularity, and focal glomerular basement membrane thickening with “double-contour” formation (Fig. 1A). No crescents or thrombi were observed. Immunofluorescence was negative for IgG, IgA, IgM, C3, C1q, and κ/λ light chains. Electron microscopy showed sparse subendothelial electron-dense deposits (Fig. 1B), and a final diagnosis of MPGN was made. Scattered lymphocytes were observed in the renal interstitium. These lymphocytes exhibited no cytological atypia. Immunohistochemical staining showed that they expressed CD20 and CD19, but were negative for CD5 and CD23, with no evidence of light-chain restriction, findings consistent with morphologically benign/reactive B cells.

During treatment, the patient developed grade 4 pneumonia complicated by sepsis (per CTCAE criteria), leading to discontinuation of therapy. After two treatment cycles, the hematologic response was assessed as stable disease. In April 2023, the patient withdrew from the study and was switched to orelabrutinib monotherapy (150 mg daily). Four months later, blood counts normalized, and both splenomegaly and lymphadenopathy resolved (Fig. 1C and D). Minimal residual disease (MRD) in peripheral blood decreased to 0.46%, and further follow-up in July 2025 showed a decline to 0.11%. The patient remains on orelabrutinib monotherapy with no treatment-related adverse events reported to date.

## Case 2: Late-onset MPGN in a patient with long-standing CLL

A 70-year-old man was diagnosed with CLL in 2007 at the age of 53 and had been under regular follow-up with stable disease for 17 years. In August 2024, he presented to the nephrology clinic with bilateral ankle edema and foamy urine. Laboratory tests revealed nephrotic-range proteinuria (24-hour urine protein: 3964 mg; uACR: 242.73 mg/mmol) with normal serum creatinine (95 µmol/L). Complete blood count showed leukocytosis (white blood cell [WBC] 129.4 × 10⁹/L) and marked lymphocytosis (absolute lymphocyte count: 118.3 × 10⁹/L). Anti-PLA2R antibodies were negative, and serum immunofixation electrophoresis did not detect monoclonal proteins. The patient was started on telmisartan for proteinuria control and referred to the hematology department. At that time, he did not meet the iwCLL criteria for treatment initiation and was managed conservatively with close monitoring.

By November 2024, his edema had worsened. Laboratory studies demonstrated a marked increase in proteinuria (24-hour urine protein: 9917 mg) and severe hypoalbuminemia (serum albumin: 18 g/L). Complete blood count showed leukocytosis (WBC 127 × 10⁹/L), hemoglobin 101 g/L, platelet count 145 × 10⁹/L, and absolute lymphocyte count of 115 × 10⁹/L. Serum creatinine was mildly elevated to 105 µmol/L. Serum immunoglobulin levels (IgG, IgA, IgM) were all decreased, but complement C3 and C4 remained within normal ranges. Bence-Jones protein was detected in the urine. Immunofixation continued to show no detectable monoclonal bands in serum. Serum β2-microglobulin was elevated to 4660 ng/mL (Table 1). Ultrasound examination revealed splenomegaly (68 × 172 mm) and generalized lymphadenopathy.

Renal biopsy was performed, which revealed marked mesangial and endocapillary proliferation under light microscopy, with diffuse glomerular basement membrane (GBM) thickening and characteristic double-contour appearance. Masson’s trichrome stain showed magenta subendothelial deposits, and Congo red staining was negative (Fig. 1E). Immunohistochemistry showed CD5⁺, CD20⁺ clonal B-cell infiltration in the interstitium, supporting CLL-related leukemic infiltration. Immunofluorescence revealed granular IgG (+++) and lambda (++) deposition along the glomerular capillary loops, with negative kappa staining. C3 (++) and C1q (+~++) were also detected (Fig. 1F). Electron microscopy confirmed abundant subepithelial and intramembranous electron-dense deposits, as well as segmental mesangial and subendothelial electron-dense deposits (Fig. 1G), consistent with a diagnosis of MPGN.

Bone marrow flow cytometry revealed 92.5% clonal mature B lymphocytes (CD19⁺, CD5⁺, CD10⁻, CD23dim⁺, CD79b⁻, FMC7⁻, CD200⁺), consistent with CLL/SLL. FISH analysis did not identify high-risk cytogenetic abnormalities, and IGHV sequencing showed a mutated status. The patient was staged as Rai III / Binet B, with a CLL-IPI score of 4 (high-risk group).

In December 2024, zanubrutinib (160 mg twice daily) was initiated. During early treatment, the WBC count increased from 127 × 10⁹/L to 181 × 10⁹/L, prompting the addition of chlorambucil (4 mg daily for 8 days). After two weeks, peripheral edema improved. By one month, serum albumin and creatinine levels normalized, 24-hour urine protein decreased to 3869 mg, WBC normalized (5.6 × 10⁹/L), and absolute lymphocyte count was 2.99 × 10⁹/L. After four months of zanubrutinib, proteinuria further declined to 1068 mg/24 h (Fig. 1H and I). To further reduce proteinuria, rituximab (375 mg/m², once monthly) was added in April 2025. After two cycles, proteinuria declined further to 267 mg/24 h. No significant treatment-related adverse events were observed other than grade 2 thrombocytopenia and occasional skin petechiae. As of the last evaluation in August 2025, the patient had achieved complete remission of nephrotic syndrome and partial hematologic remission of CLL.

## Discussion

Although renal complications in CLL are rare and often under-recognized, they encompass a broad spectrum of pathologies. Among them, MPGN is one of the most frequently reported glomerular lesions. It represents a histopathological pattern rather than specific disease entity, driven primarily by immune complex deposition or dysregulation of the alternative complement pathway. The former requires thorough evaluation for potential infections, autoimmune diseases, or monoclonal gammopathies, while the latter is further classified into dense deposit disease (DDD) and C3 glomerulonephritis (C3GN) based on electron microscopy findings [2, 4, 7].

In this report, we present two cases of CLL-associated MPGN, each reflecting a distinct clinical course. One case involved simultaneous diagnosis of CLL and MPGN, while the other developed MPGN as a late complication 17 years after CLL diagnosis.

Monoclonal gammopathy is present in 5–10% of patients with CLL, and approximately 1% of these patients develop glomerular disease, with MPGN being the most common pathological type [3]. Monoclonal proteins derived from the clonal B-cell population in CLL can induce glomerular injury either through immune complex deposition or via dysregulation of the alternative complement pathway [2, 7].

In Case 1, although immunohistochemistry of the renal biopsy showed no evidence of CD5⁺CD23⁺ CLL cell infiltration, the overall hematologic and renal response to CLL-directed therapy supports a potential pathogenic link between the underlying lymphoproliferative disorder and kidney injury. In our patient, the presence of an IgG-κ monoclonal component, isolated low serum C3, and electron-dense deposits without detectable immunoglobulin staining on immunofluorescence supports a mechanism of alternative complement pathway activation rather than classic immune complex deposition. This constellation of findings is consistent with the spectrum of complement-mediated membranoproliferative glomerulonephritis within the context of monoclonal gammopathy of renal significance [8]. Taken together, the evidence indicates that the renal injury in this patient was likely mediated by a nephrotoxic monoclonal protein secreted by the CLL clone, which triggered glomerular damage through complement pathway activation.

Case 2 exhibited classic immune complex–mediated MPGN. Immunofluorescence showed strong granular deposition of IgG, C3, and C1q along the glomerular capillary walls, accompanied by CLL cell infiltration in the interstitium. Combined immunopathologic and ultrastructural findings supported the pathogenesis of either circulating immune complexes induced by tumor-associated antigens or in situ formation of immune complexes derived from locally secreted immunoglobulins by infiltrating CLL cells.

These cases highlight the heterogeneity of CLL-associated MPGN and the indispensable role of renal biopsy in confirming the diagnosis and underlying mechanism. In CLL patients presenting with new-onset proteinuria or renal dysfunction—especially with concurrent immune abnormalities or monoclonal proteins—early renal biopsy is crucial to guide management. According to iwCLL guidelines, renal parenchymal involvement alone can serve as an indication to initiate CLL-directed therapy [1].

The treatment of MPGN hinges on addressing the underlying disease. Previous reports describe the use of corticosteroids, chemotherapy (e.g., chlorambucil, cyclophosphamide), and anti-CD20 monoclonal antibodies (e.g., rituximab) in CLL-associated MPGN, but outcomes have been variable and often limited by poor tolerability [2, 5, 7]. Recently, BTKis have transformed the treatment landscape of CLL, demonstrating superior efficacy and tolerability in high-risk subgroups such as those with unmutated IGHV or TP53 mutations. Compared with ibrutinib, next-generation BTKis such as zanubrutinib and orelabrutinib offer improved selectivity and reduced adverse effects, and are now recommended as first-line therapies [9, 10]. Building on their established role in CLL, our cases suggest that next-generation BTKis may represent an effective therapeutic option for CLL-associated renal injury. It is crucial to emphasize that treatment responses can be highly disease-specific. This is illustrated by the contrasting experience in Waldenström macroglobulinemia with AL amyloidosis, where the first-generation BTKi ibrutinib has shown limited efficacy in reducing proteinuria despite hematologic response, and its use is often discouraged due to toxicity concerns [11]. This disparity underscores that the efficacy and safety profile of a drug class cannot be extrapolated across different lymphoproliferative disorders, and highlights the need for disease- and mechanism-driven therapeutic approaches. Our experience with next-generation, highly selective BTKis in CLL-MPGN provides a new therapeutic option for this condition.

Anti-CD20 monoclonal antibodies (e.g., rituximab) may serve as adjuncts to BTKi-based regimens, particularly in patients requiring rapid tumor debulking or enhanced renal remission. However, their use should be carefully weighed against the increased risk of infection, especially in immunocompromised or renally impaired patients.

In our cases, both patients received BTKi-based treatment with favorable outcomes. Case 1 initially received rituximab combined with chemotherapy and achieved rapid renal remission but developed severe infection with persistent CLL activity. Switching to orelabrutinib resulted in hematologic improvement. In Case 2, zanubrutinib monotherapy significantly improved proteinuria and hematologic parameters, and subsequent addition of rituximab let to a further reduction in urinary protein, ultimately achieving dual remission of CLL and MPGN.

In summary, these two cases illustrate the clinical heterogeneity of CLL-associated MPGN and underscore the diagnostic value of renal biopsy in guiding individualized treatment. The BTKi-based regimens demonstrated efficacy and well tolerated in our patients. Further studies are needed to better define optimal management strategies in this rare patient population.

**Table 1:** Baseline characteristics at treatment initiation

	Case 1	Case 2
Age (years) / Sex	29 / Male	70 / Male
CLL presentation	First manifestation	17 years after diagnosis
White blood cell count (× 10^9^/L)	4.29	127
Absolute lymphocyte count (× 10^9^/L)	3.56	115
Hemoglobin (g/L)	67	101
Platelet (× 10^9^/L)	43	145
Serum albumin (g/L)	25	18
Serum creatinine (µmol/L)	103	105
Uric acid (µmol/L)	709	406
eGFR (mL/min/1.73 m²)	84.2	61.7
Serum β₂-microglobulin (ng/mL)	6038	4660
Proteinuria (mg/24 h)	7643	9917
uACR (0-2.5 mg/mmol)	209.9	242.73
Complement C3 (0.74–1.4 g/L)	0.68	0.78
Complement C4 (0.1–0.4 g/L)	0.16	0.28
IgG (8.6–17.4 g/L)	4.55	2.91
IgA (1–4.2 g/L)	1.05	0.49
IgM (0.3–2.2 g/L)	0.09	0.18
Serum monoclonal protein	IgG-κ (1.2 g/L)	Negative
Bence Jones proteinuria	κ-type Bence Jones proteinuria	Positive
ANCA	Negative	Negative
Anti-PLA2R Ab	Negative	Negative
Serum viral testing*	Negative	Negative
FISH (TP53, ATM, CSP12, RB1, D13525)	Negative	Negative
IGHV status	Unmutated	Mutated
Rai stage	IV	III
Binet stage	C	B
CLL-IPI score	5	4

Viral panel* (Hepatitis B surface antigen, Hepatitis C virus antibody, Human immunodeficiency virus antibody and Treponema pallidum antibody)

Abbreviations：CLL, Chronic lymphocytic leukemia; eGFR, estimated glomerular filtration rate; uACR, Urinary Albumin-to-Creatinine Ratio; ANCA, antineutrophilic cytoplasmic antibody; PLA2RAb, phospholipase A2 receptor antibody

## Data Availability

The data that support the findings of this study are available from the corresponding author upon reasonable request.
